# Thematic issue of the Second combined Bio-ontologies and Phenotypes Workshop

**DOI:** 10.1186/s13326-016-0108-7

**Published:** 2016-12-12

**Authors:** Karin Verspoor, Anika Oellrich, Nigel Collier, Tudor Groza, Philippe Rocca-Serra, Larisa Soldatova, Michel Dumontier, Nigam Shah

**Affiliations:** 1 Department of Computing and Information Systems, The University of Melbourne, Melbourne, VIC Australia; 2 MRC Social, Genetic & Developmental Psychiatry Centre (SGDP), King’s College London, London, SE5 8AF UK; 3 The Language Technology Lab, Department of Theoretical and Applied Linguistics, University of Cambridge, Cambridge, UK; 4 Centre for Clinical Genomics, Garvan Institute of Medical Research, Sydney, NSW Australia; 5 St Vincent’s Clinical School, Faculty of Medicine, University of New South Wales, Sydney, Australia; 6 University of Oxford e-Research Centre, 7 Keble Road, OX1 3QG Oxford, UK; 7 Brunel University London, London, UK; 8 Stanford University, Stanford, CA USA

## Abstract

This special issue covers selected papers from the 18th Bio-Ontologies Special Interest Group meeting and Phenotype Day, which took place at the Intelligent Systems for Molecular Biology (ISMB) conference in Dublin in 2015. The papers presented in this collection range from descriptions of software tools supporting ontology development and annotation of objects with ontology terms, to applications of text mining for structured relation extraction involving diseases and phenotypes, to detailed proposals for new ontologies and mapping of existing ontologies. Together, the papers consider a range of representational issues in bio-ontology development, and demonstrate the applicability of bio-ontologies to support biological and clinical knowledge-based decision making and analysis.

The full set of papers in the Thematic Issue is available at http://www.biomedcentral.com/collections/sig.

## Introduction

This special issue originates from the papers presented in a 2-day meeting, combining the Bio-Ontologies SIG (Special Interest Group) meeting with a phenotype-focused Phenotype Day, held at the Intelligent Systems for Molecular Biology (ISMB) conference in Dublin, Ireland in 2015. This was the second combined event, following on from a successful event in 2014 [1]. The papers that feature in this special issue are a selection of submissions to the meeting that were extended and submitted for consideration by the journal. All papers were substantially revised from the original SIG meeting papers, and underwent the standard journal peer review process.

The Bio-Ontologies meeting, as in years past, invited presentation and discussion of research across a broad scope, encompassing the organization and dissemination of knowledge in biomedicine and any aspect of application of ontologies in life sciences research. There were 14 submissions to the Bio-Ontologies SIG meeting, and 12 were accepted to the workshop, including six short papers and six flash updates. Of these, four were extended for this special issue.

Phenotype Day brought together researchers from a large number of different domains to discuss aspects of representation, integration and dissemination of phenotype data across domains and disciplines. There were 15 submissions in total to Phenotype Day, with 11 accepted to the workshop (one full paper, six short papers, one position paper, three posters). Of these, five appear in this special issue.

## Summary of selected papers

In correspondence with the broad selection of topics represented at the Bio-Ontologies Special Interest Group meeting, the papers selected for this special issue also cover this range of topics. The areas of interest included not only the creation, further development and integration of existing ontologies, but also their application in phenomics research. Application areas include the representation and integration of model organism databases as well as text mining of the scientific literature or clinically relevant documents, such as electronic health records.

### Summary of papers from bio-ontologies

Two papers in this special issue address issues core to the construction of ontologies for management of biological information. Vita et al. [2] directly propose an extension to a previously-proposed Major Histocompatibility Complex (MHC) Restriction ontology called MaHCO [3], constructed with the assistance of ontology design patterns, while Jupp et al. [4] introduce a web-based tool to ease authoring of ontologies with built-in support for enforcing precisely such design patterns.

Specifically, the ontology proposed by Vita et al. [2] aims to enable representation of MHC molecules in the Immune Epitope DataBase (IEDB), in terms of their relation to immunological experiments. These molecules play an important role in the adaptive immune system, and because of their wide variation and broad relevance, pose a challenge to knowledge representation. The enriched MHC ontology enables logical querying of MHC molecules, in terms of a protein complex of two chains, and includes the details of their locus, haplotype and/or serotype, as well as the haplotype of the host species. Finally, the experimental evidence for the MHC restriction is also modelled. The authors have provided users of the IEDB the capability to search complex relationships among MHC genes and MHC restrictions, in terms of standard ontology identifiers wherever possible.

In their software article, Jupp et al. [4] introduce the Webulous application suite, including an add-on application for Google Sheets that allows population of ontology design templates with content, and demonstrate it with a case study using the Experimental Factor Ontology (EFO). This software allows addition of ontology content in bulk, while ensuring consistency of that content. It includes access to BioPortal services [5] that allow users to search for existing ontology terms to facilitate ontology integration and reuse. The templates themselves allow automatic creation of relations or assertions from data entered into a spreadsheet, using consistent transformation of the data to OWL axioms. In short, it supports large-scale ontology development with the assistance of domain experts who may not themselves be ontology experts.

Webulous is used to create terms in EFO for the work described by Sarntivijai et al. [6]. In the context of the Centre for Therapeutic Target Validation (CTTV), they aim to represent disease-phenotype associations, with the objective of linking rare and common diseases to enable identification of potential therapeutic (drug) targets. A particular representational challenge tackled in this research is to capture phenotypes that are only *sometimes associated* to a disease, to reflect that not all relevant phenotypes will be present in every presentation. This is done through the use of a generic association model OBAN (Open Biomedical AssociatioN) which allows qualification of association with evidence and, eventually, frequency. The authors describe the use of text mining of the literature to identify candidate disease-phenotype associations that are curated and transformed into the OBAN model using EFO.

Leung and Dumontier [7] similarly apply text mining in the context of disease associations, in their case considering drug-disease associations as extracted from drug structured product labels. The identified associations are compared to the clinical practice guidelines, with the finding that there is not a large overlap between the disease indications for drugs in their structured labels, and the indications for those same drugs in clinical practice guidelines. The authors did find that using taxonomic relationships among drugs did improve the overlap, but a substantial gap remained. The study raises concerns about the inconsistent evidence between these drug-related information sources and has implications for clinical decision making in evidence-based practice.

### Summary of papers from Phenotype Day

Bello and colleagues report in “Inferring Gene-to-Phenotype and Gene-to-Disease Relationships at Mouse Genome Informatics: Challenges and Solutions” [8] on an algorithm for the assignment of gene-phenotype and gene-disease association from the existing genotype-phenotype links contained in the Mouse Genome Informatics (MGI) database. The algorithm has been applied to the existing wealth of data in this database to the effect that 2100 mouse markers could be linked to human disease and 16,000 mouse markers could be linked to phenotypes. The resulting gene-phenotype and gene-disease associations are provided as part of the database’s web pages and can be downloaded by interested parties.

In “Interoperability between phenotypes in research and healthcare terminologies - Investigating partial mappings between HPO and SNOMED CT” [9], Dhombres and colleagues report about their investigations to determine partial alignments between both the Human Phenotype Ontology (HPO) and SNOMED CT using modifier terms and HPO’s subsumption relations. Using the suggested approach, the authors identified partial mappings for 92% of the investigated HPO terms. 30% out of these 92% partial mappings correspond to equivalence statements, while the remaining 60% follow a next-best approach to allow for traversing between both ontologies.

Mowery et al. in “Extracting a Stroke Phenotype Risk Factor from Veteran Health Administration Clinical Reports: An Information Content Analysis” conducted experiments to investigate the report of a stenosis phenotype in relation to stroke in radiology and text integration utility notes [10]. These notes were gathered from the Veteran Health Administration electronic health records. The authors analyse sections and pure textual representations in both types of records using pyConText. The results show that there are differences in the performance of stenosis identification and the location of reporting for both types of note. Yet the authors conclude that pyConText can still be used to filter chart reports into significant and no/insignificant stenosis findings for the data from the Veteran Health Administration, facilitating further studies on effectiveness of stroke prevention.

Tudose et al. present in “PhenoImageShare: An image annotation and query infrastructure” [11] a phenotype annotation infrastructure for image data. Images are imported from four different resources, leveraging ontology annotations from the original repository. Furthermore, images can be manually annotated using a variety of ontologies, such as UBERON or the Mammalian Phenotype Ontology (MP). The annotation service is independent from species and image data. PhenoImageShare holds to date ~118 k images (retrieved from mouse and fly databases) associated to anatomical or phenotype concepts (so called regions of interest). The phenotype image data can be accessed either via a web interface or an API.

The manuscript “Reporting phenotypes in mouse models when considering body size as a potential confounder” by Oellrich and colleagues [12] investigates the challenges surrounding confounding variables in experimental studies associating genotypes and phenotypes. The authors provide a case study based on the experimental results released by the International Mouse Phenotyping Consortium (IMPC) and further discuss the limitations of current ontological representation to report on confounding effects. The authors conclude that further discussion is needed within the community to derive a community-approved representation and dissemination of confounders in genotype-phenotype association studies.

## Conclusions

The storage, retrieval, and analysis of ever-growing biological information is complicated by the complexity and diversity of that information. To the extent that consistency in representation of this information can be achieved through the use of a common terminology and validated relationships, and that strategies for modeling rare, confounded, or highly contextual relationships can be developed, it is possible to make progress on making that information findable and available for further investigation. The papers in this thematic issue contribute to those goals, both by addressing the foundational issues of representational expressivity as well as consistency of use of bio-ontologies, and by demonstrating the application of such structured representations to support inference from biological or biomedical data, on tasks ranging from determination of stroke risk factors and predictions of novel gene-disease associations. The papers raise some important challenges yet add to the body of research that establishes the promise of improved biomedical information access and analysis.
